# Supplementary material for: An electrogenic redox loop in sulfate reduction reveals a likely widespread mechanism of energy conservation
Source: Nat Commun. 2018 Dec 21;9:5448. doi: 10.1038/s41467-018-07839-x (PMC6303296; doi:10.1038/s41467-018-07839-x)
Supplement: Supplementary file 1 — Supplementary Information [file 41467_2018_7839_MOESM1_ESM.pdf]

# Supplementary Information

## An electrogenic redox loop in sulfate reduction reveals a likely widespread mechanism of energy conservation

Américo G. Duarte[a], Teresa Catarino[a,b], Gaye F. White[c], Diana Lousa[a], Sinje Neukirchen[d], Cláudio M. Soares[a], Filipa L. Sousa[d], Thomas A. Clarke[c] and Inês A. C. Pereira*[a]

Author affiliations:

[a]Instituto de Tecnologia Química e Biológica António Xavier, Universidade Nova de Lisboa, Av. da República, 2780-157 Oeiras, Portugal

[b]Departamento de Química, Faculdade de Ciências e Tecnologia, Universidade Nova de Lisboa, 2829-516 Caparica, Portugal

[c]Centre for Molecular and Structural Biochemistry, School of Biological Sciences and School of Chemistry, University of East Anglia, Norwich, NR4 7TJ, UK

[d]Division of Archaea Biology and Ecogenomics, Department of Ecogenomics and Systems Biology, University of Vienna, Althanstrasse 14 UZA I, 1090 Vienna, Austria

*corresponding author: ipereira@itqb.unl.pt

Supplementary Table 1. Details of the QrcD/NrfD/PsrC proteins.

| Protein | Predicted Transmembrane helices | FeS partner | Type of catalytic subunit | Catalytic subunit location | Pathway | $E^{o\prime}$ (mV) | Ref. |
|---|---|---|---|---|---|---|---|
| **Quinone reductases** | | | | | | | |
| **QrcD** | 10 | QrcC | MHCC+Mo/W-*bis*PGD | Periplasm | $H_2$/formate oxidation | $H^+/H_2$ -412 | [1] |
| **HybB** | 10 | HybA | NiFe | Periplasm | $H_2$ oxidation | $H^+/H_2$ -412 | [2] |
| **SoeC** | 8 | SoeB | Mo/W-*bis*PGD | Cytoplasm | Sulfite oxidation | $SO_4^{2-}/SO_3^{2-}$ -516 | [3] |
| **Quinol oxidases** | | | | | | | |
| **NrfD** | 8 | NrfC | MHCC | Periplasm | Nitrite reduction | $NO_3^{2-}/NH_4^+$ +340 | [4] |
| **ActC** | 10 | ActB | MHCC+Mo/W-*bis*PGD | Periplasm | $O_2$ reduction | $O_2/H_2O$ +820 | [5,6] |
| **DmsC** | 8 | DmsB | Mo/W-*bis*PGD | Periplasm | DMSO reduction | DMSO/DMS +160 | [7] |
| **ArrC** | 8 | ArrB | Mo/W-*bis*PGD | Periplasm | $As^V$ reduction | $As^V/As^{III}$ +60 | [8] |
| **TtrC** | 9 | TtrB | Mo/W-*bis*PGD | Periplasm | Tetrathionate reduction | $S_4O_6^{2-}/S_2O_3^{2-}$ +24 | [9] |
| **DsrP** | 10 | DsrO | MHCC | Periplasm | Sulfite reduction | $HSO_3^{2-}/HS^-$ -116 | [10] |
| **MccD** | 8 | MccC | MHCC | Periplasm | Sulfite reduction | $HSO_3^{2-}/HS^-$ -116 | [11] |
| **PsrC** | 8 | PsrB | Mo/W-*bis*PGD | Periplasm | Polysulfide reduction | $S_4^{2-}/S^{2-}$ -260 | [12,13] |
| **SreC** | 10 | SreB | Mo/W-*bis*PGD | Periplasm | Sulfur reduction | $S^0/S^{2-}$ -275 | [14] |
| **Uncertain** | | | | | | | |
| **HmcC** | 10 | HmcD | MHCC | Periplasm | ? | ? | [15] |

MHCC – multiheme *c*-type cytochrome

Mo/W-*bis*PGD – molybdenum or tungsten bis-pterin guanosine dinucleotide cofactor containing proteins

NiFe – nickel-iron hydrogenase catalytic subunit

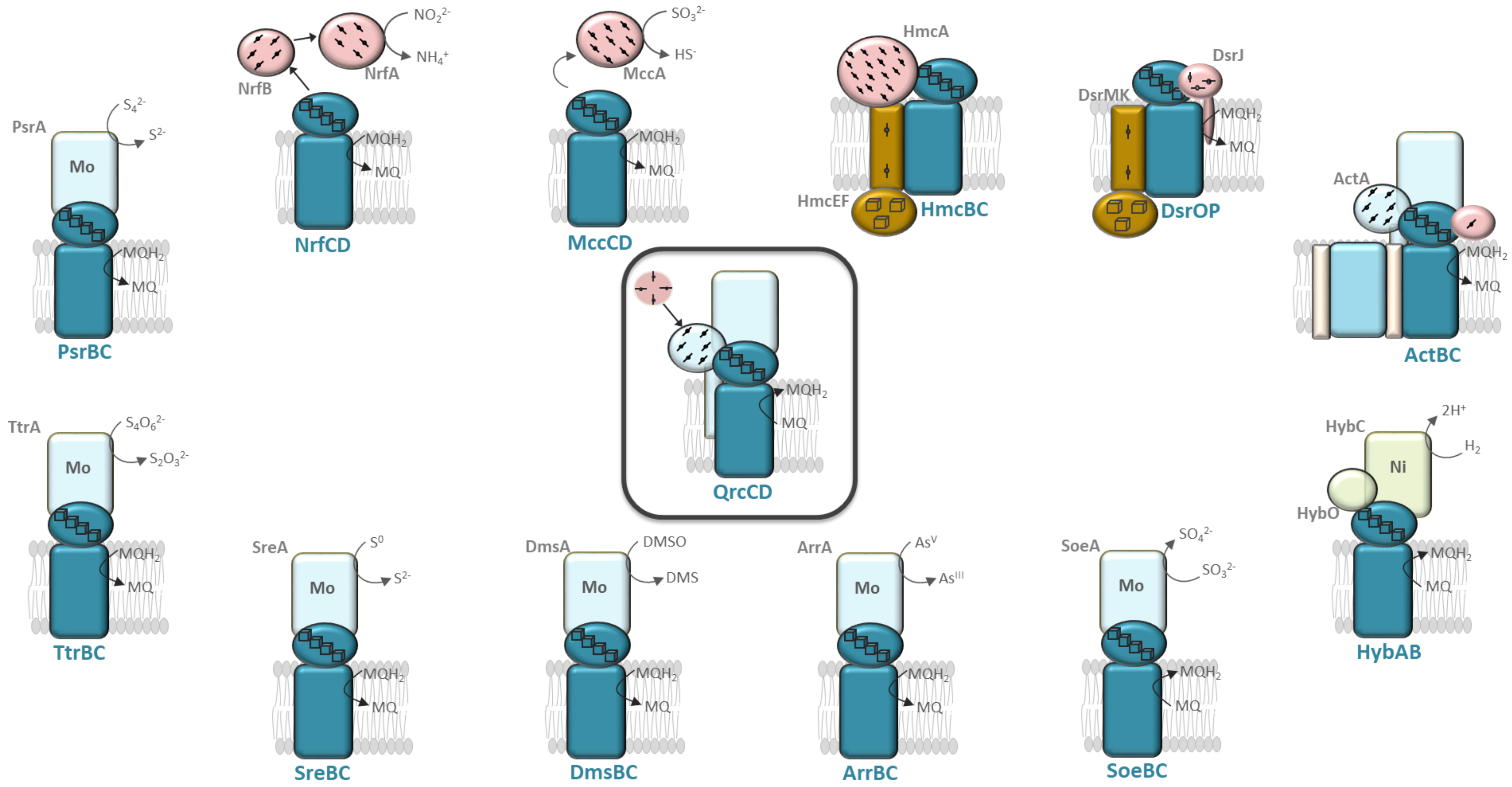


**Supplementary Fig. 1 l Schematic representation of the respiratory complexes.** Complexes described in Table 1, which contain the QrcCD-like dimeric redox module (in dark blue), are presented.

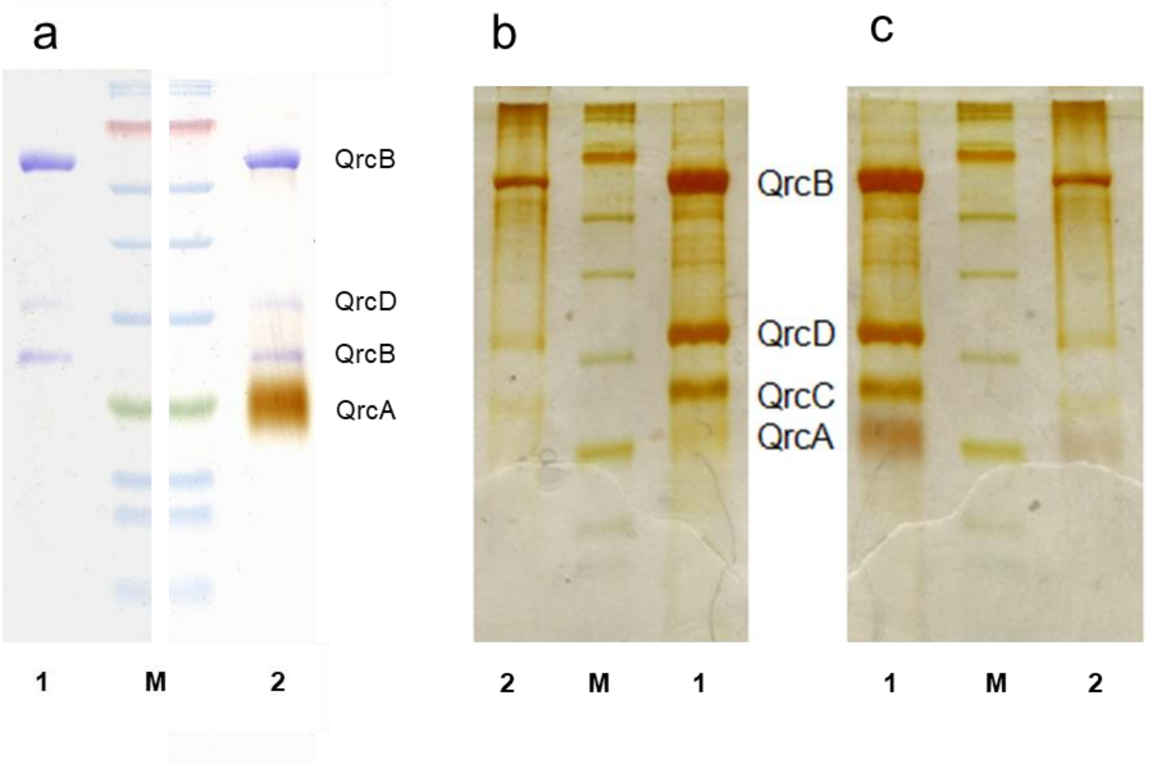


**Supplementary Fig. 2 l SDS-PAGE of the QrcABCD complex**. a) as-isolated QrcABCD complex, 1 - stained with Coomassie Blue, and 2 - also stained for hemes. b) 12% SDS-Tricine gel stained with silver and c) also stained for hemes. 1 – as-isolated QrcABCD complex, and 2 - QrcABCD complex after liposome reconstitution and precipitation in ice cold acetone. M-NZYColour Protein Marker II (Nzytech),

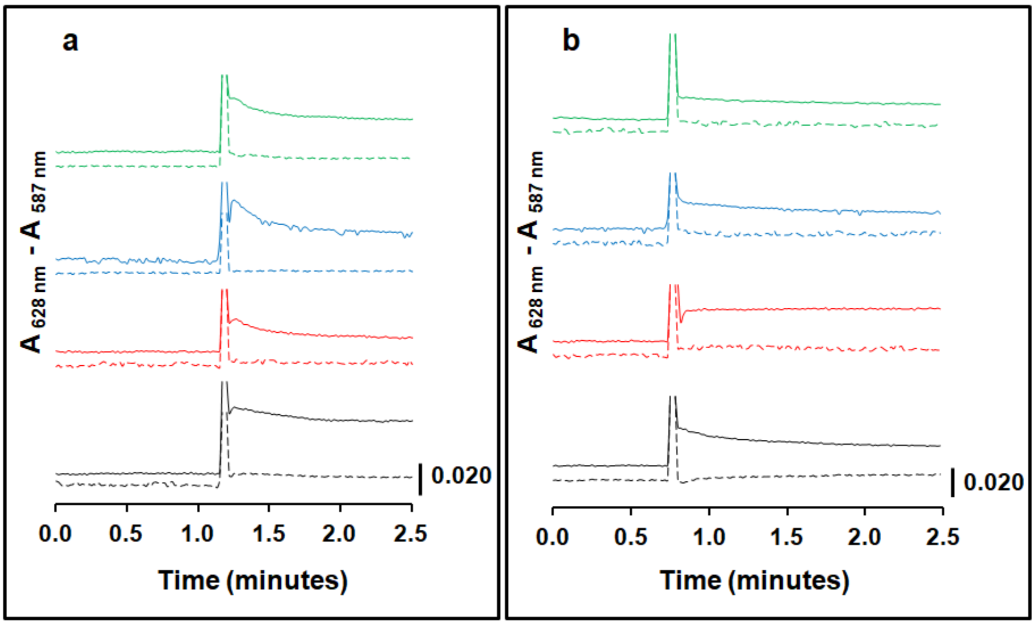


**Supplementary Fig. 3. Evaluation of liposome integrity.** Integrity was evaluated by generation of a membrane potential in valinomycin permeabilised liposomes. (a) Liposomes prepared without or (b) with phenol red in the inner cavity. Full lines – addition of a KCl saturated solution and dashed lines – addition of the same volume of buffer. Empty liposomes (grey); Qrc proteoliposomes (red); MK4 liposomes (blue) and Qrc/MK4 proteoliposomes (green).

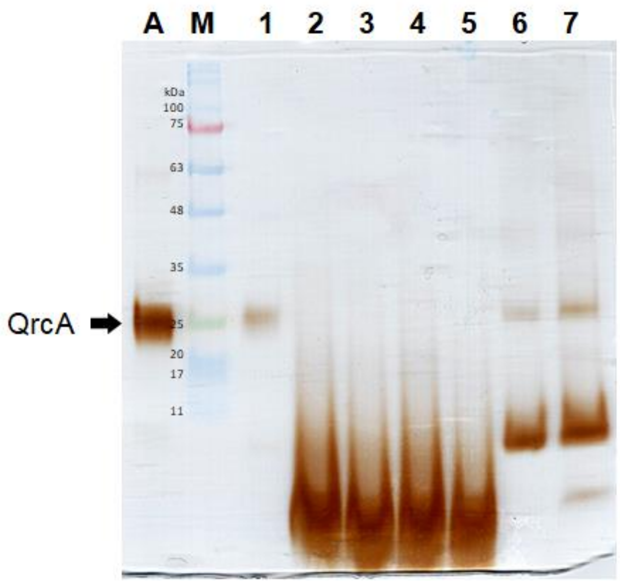


**Supplementary Fig. 4. Evaluation of QrcABCD orientation in proteoliposomes.** QrcABCD proteoliposomes (1-5) were treated with proteinase K and analyzed by 10% SDS-PAGE Tricine gel, stained for heme. Lanes: A-QrcABCD (as purified); M-NZYColour Protein Marker II (Nzytech); 1- before proteinase K incubation; 2-5 incubated with proteinase K for 1, 2, 5 and 10 minutes, respectively; 6, 7- liposomes containing horse cytochrome *c* inside not incubated (6) or incubated (7) with proteinase K, 10 minutes at 37 ºC.

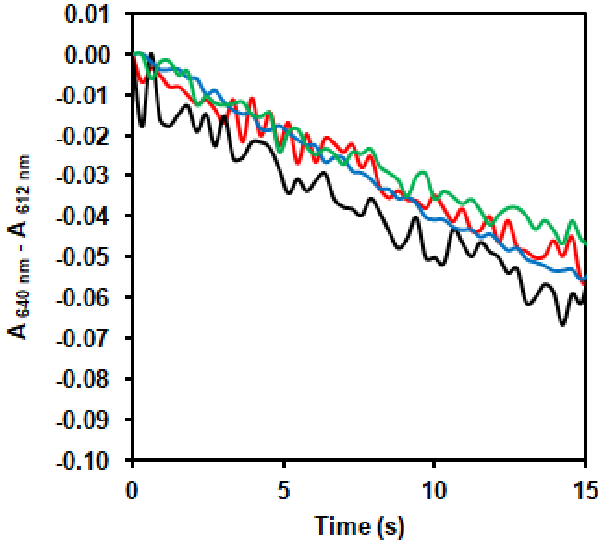


**Supplementary Fig. 5. Membrane potential dissipation by valinomycin.** Absorbance ($A_{640nm}$ - $A_{612nm}$) after mixture of reduced TpI*c*$_3$ with valinomycin permeabilised liposomes, in the presence of oxonol V. Empty liposomes (black); Qrc proteoliposomes (red); MK4 liposomes (blue) and Qrc/MK4 proteoliposomes (green).

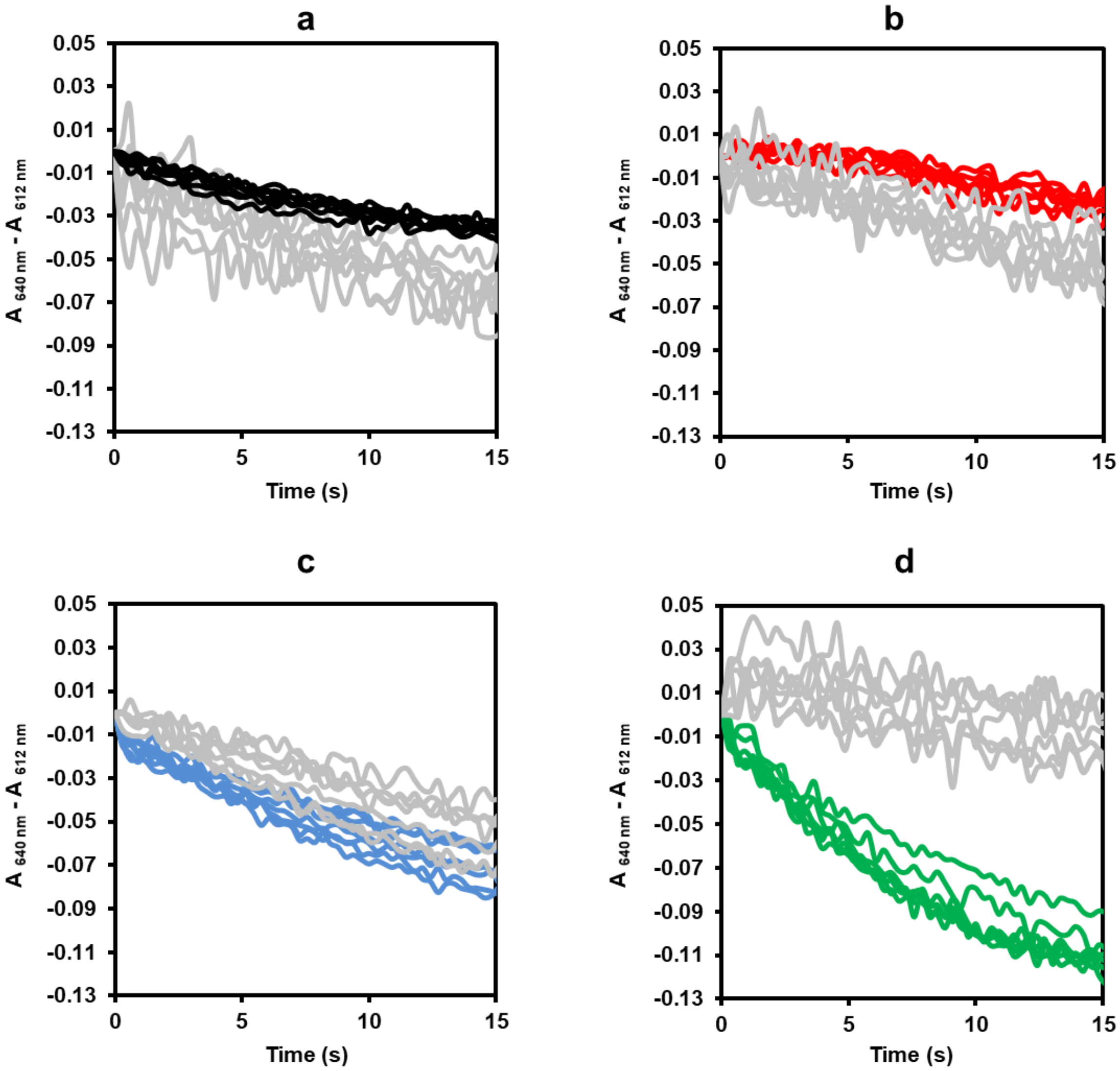


**Supplementary Fig. 6 – | Experimental raw data for changes in membrane potential.** Absorbance ($A_{640nm}$ - $A_{612nm}$) of oxonol V after mixture of reduced TpIc$_3$ with liposomes lacking valinomycin (colored lines) and with valinomycin-permeabilised liposomes (grey lines). a) Empty liposomes (black); b) Qrc proteoliposomes (red); c) MK4-liposomes (blue) and d) Qrc/MK4-proteoliposomes (green).

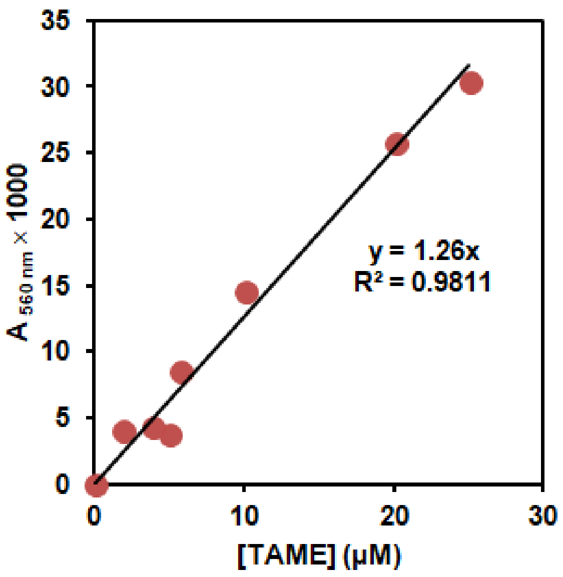


**Supplementary Fig. 7. Phenol red calibration curve.** Calibration curve performed in the presence of phenol red with tryptic hydrolysis of N-α-tosyl-L-arginyl-O-methylester (TAME), used to convert phenol red absorbance at 560 nm to proton concentration. For experimental details see online methods.

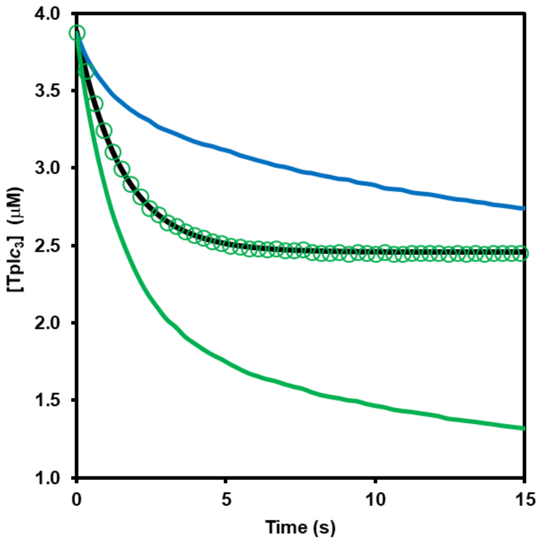


**Supplementary Fig. 8. Concentration of reduced TpI$c_3$ in MK4/Qrc containing liposomes.** The experimental data presented in Fig. 1b (absorbance at 552 nm) was converted to TpI$c_3$ concentration (μM). Kinetic trace subtraction (green circles) of MK4-liposomes trace (blue) from the MK4/Qrc proteoliposomes (green). Single exponential fit (A exp $^{(-k\times t)}$ + C) (black line) over the experimental data, with A=-1.43 $\pm$ 0.14 μM, k=0.65 $\pm$ 0.05, C=2.46 $\pm$ 0.20 μM. The experiments contained 6.3 nM of QrcABCD in the assay, giving a $K_{cat}$ of 147 $s^{-1}$.

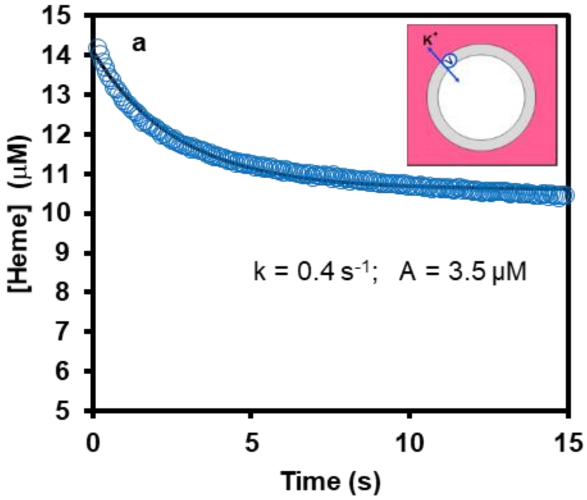


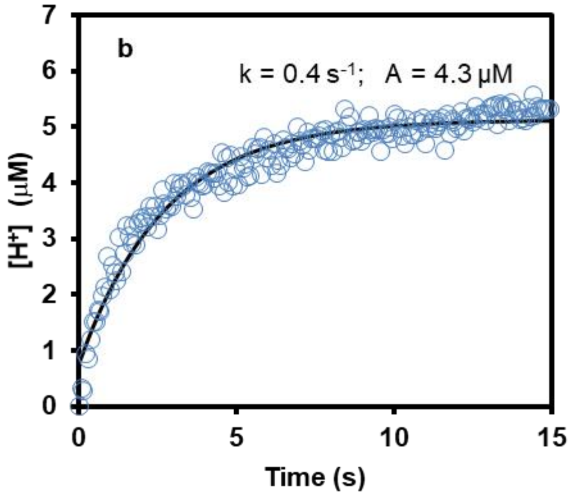


**Fig. 9 l Electron and proton transfer experiments with phenol red in the outer solution.** a) Ferrous heme concentration and b) proton concentration after mixture of reduced Tpl$c_3$ with valinomycin-permeabilised MK4-liposomes (blue dots). The black lines correspond to single exponential fits with the following equations: Heme oxidation: $3.5 \times \exp^{(-0.4 \times t)}+10.6$; Proton consumption: $-4.3 \times \exp^{(-0.4 \times t)}+ 5.1$.

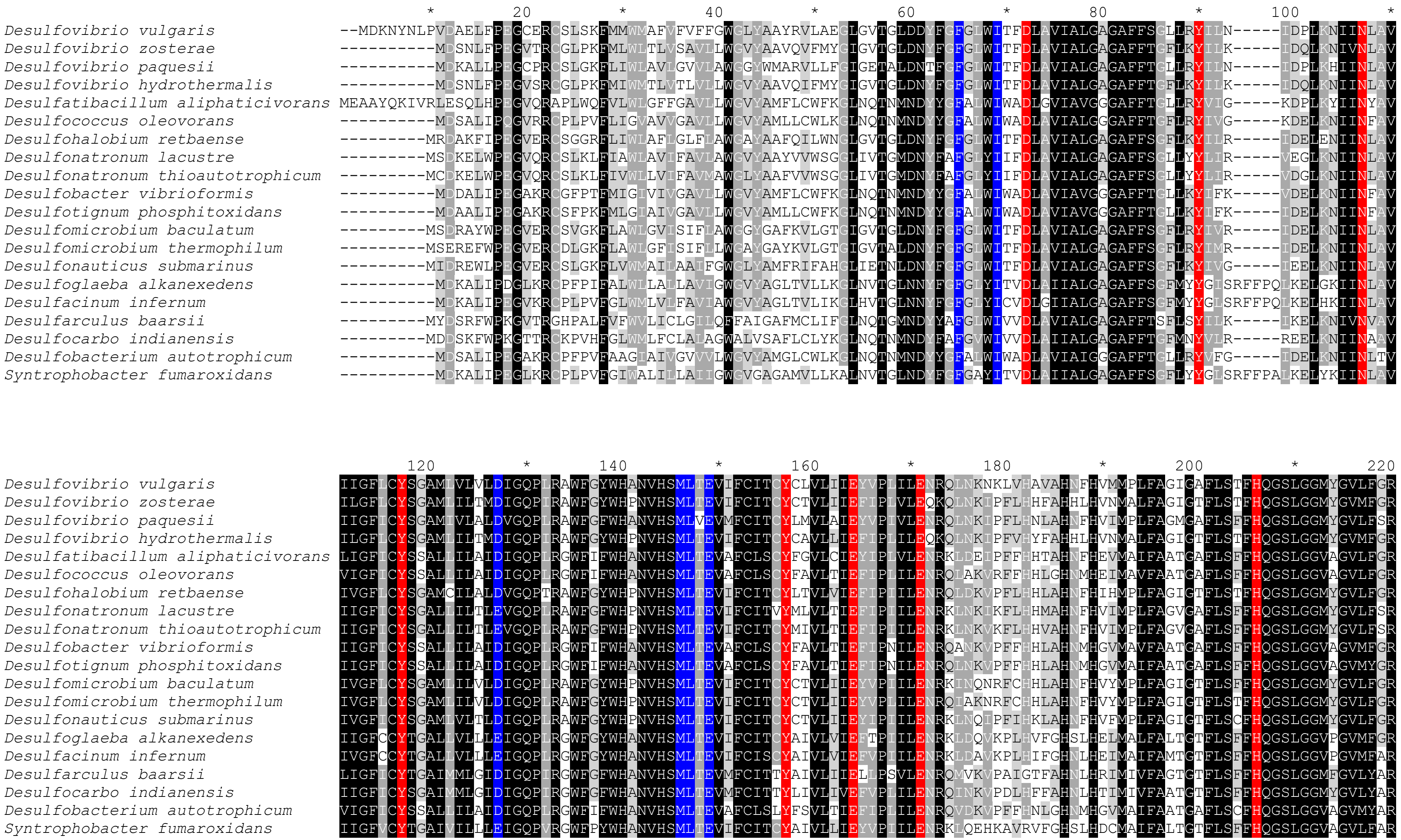
Desulfovibrio vulgaris
Desulfovibrio zosterae
Desulfovibrio paquesii
Desulfovibrio hydrothermalis
Desulfatibacillum aliphaticivorans
Desulfococcus oleovorans
Desulfohalobium retbaense
Desulfonatronum lacustre
Desulfonatronum thioautotrophicum
Desulfobacter vibrioformis
Desulfotignum phosphitoxidans
Desulfomicrobium baculatum
Desulfomicrobium thermophilum
Desulfonauticus submarinus
Desulfoglaeba alkanexedens
Desulfacinum infernum
Desulfarculus baarsii
Desulfocarbo indianensis
Desulfobacterium autotrophicum
Syntrophobacter fumaroxidans

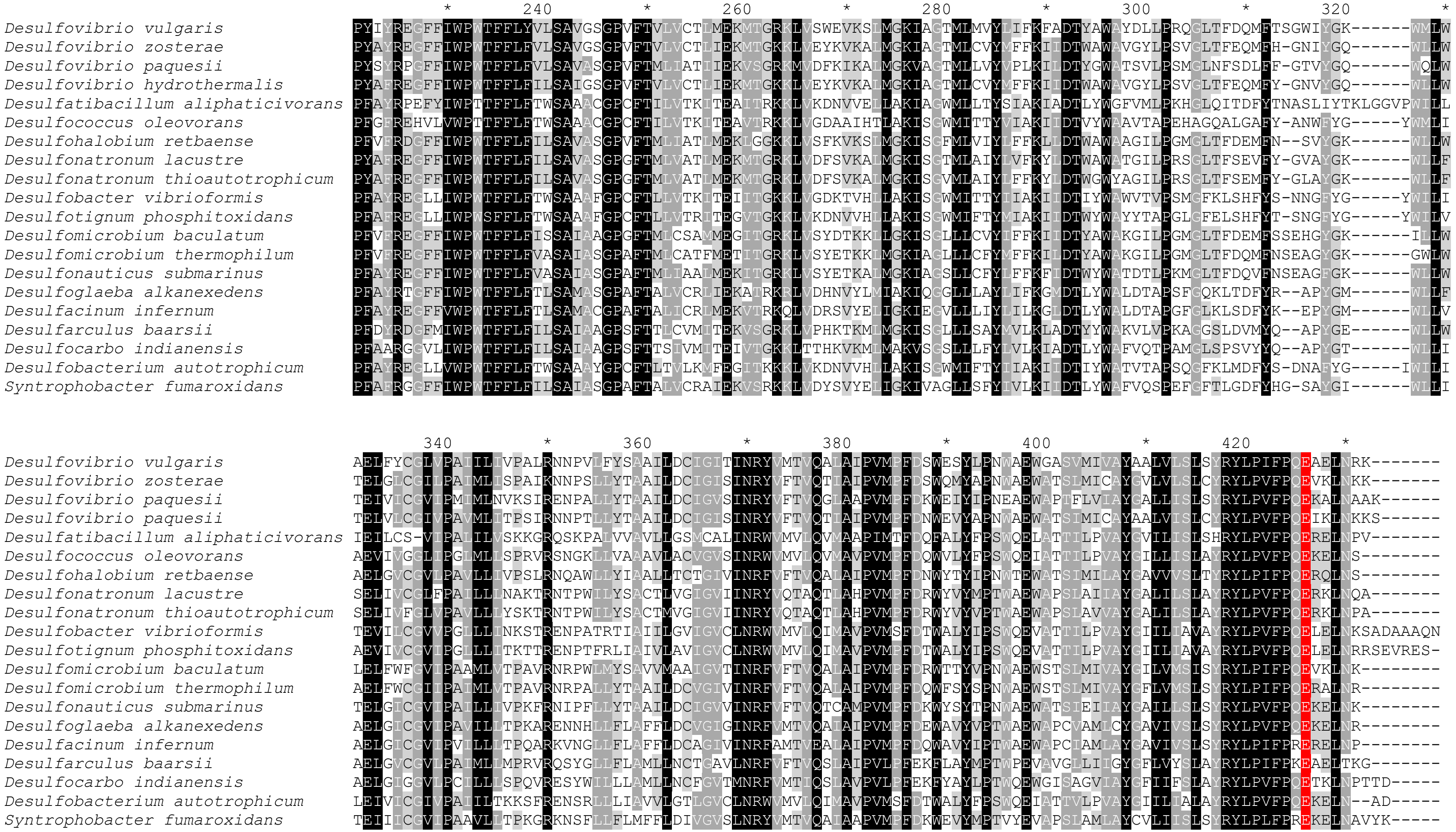


**Supplementary Fig. 10. QrcD multiple sequence alignment.** The QrcD sequences were downloaded from the Joint Genome Institute (https://jgi.doe.gov/) and the aligned with ClustalX 2.1. Conserved residues for the predicted quinone binding site and proton pathway are coloured in blue and red, respectively.

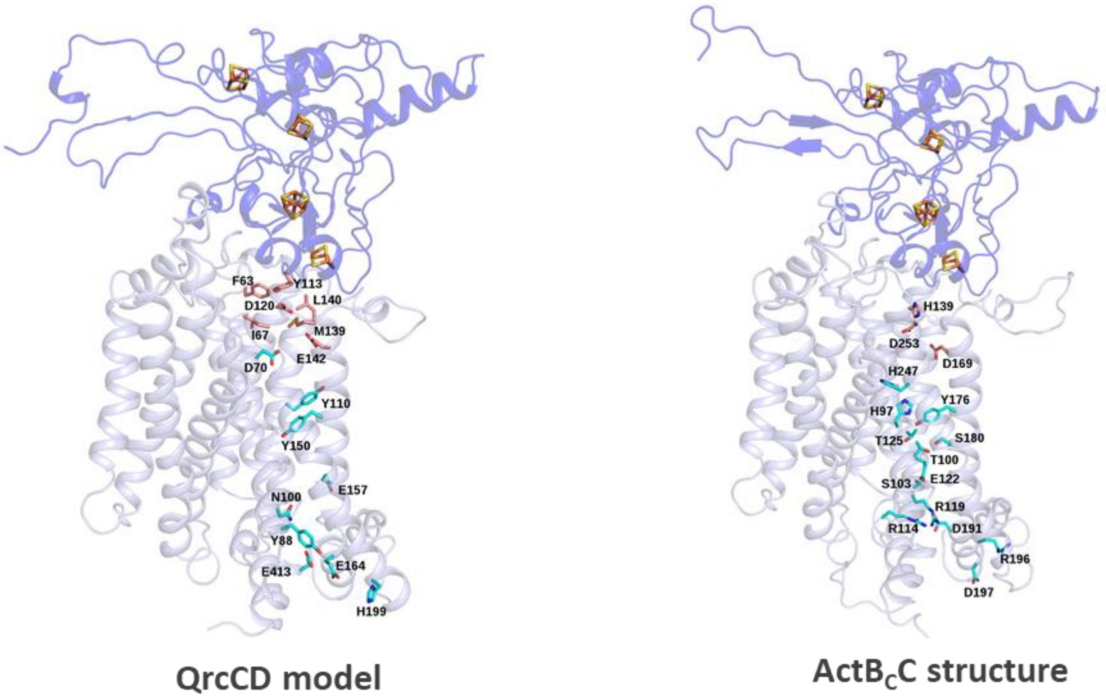


**Supplementary Fig. 11: Comparison of the QrcCD model and $ActB_CC$ structure.** The proposed residues involved in quinone-binding and proton channels (only the cytoplasmic channel for ActC) are highlighted using sticks with carbons colored in orange and cyan, respectively.

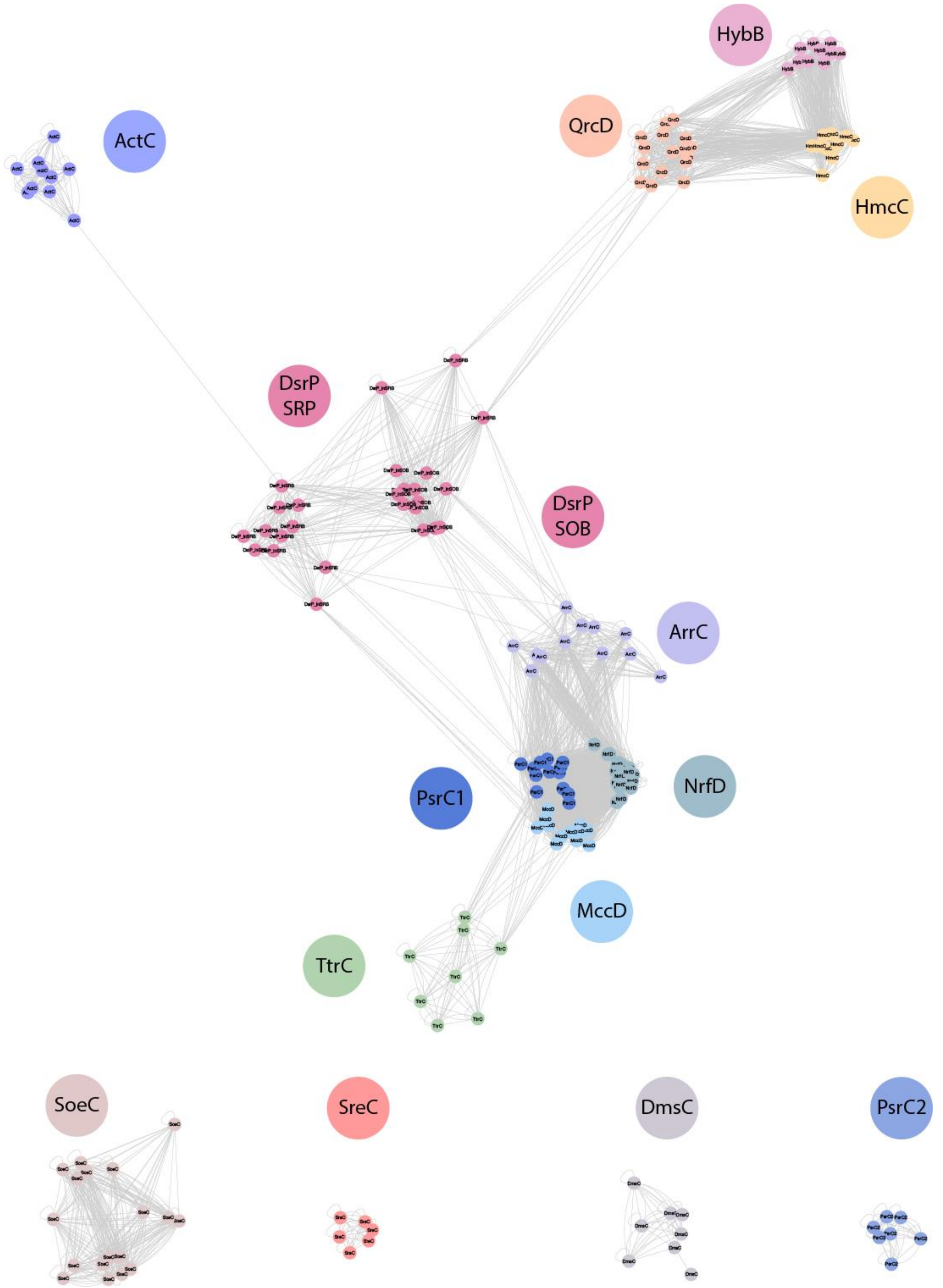


**Supplementary Fig. 12: Similarity network representation of the QrcD/PsrC/NrfD family.** Each circle (node) represents a protein sequence and the grey lines (edges) connecting the circles represent the identity between two sequences. Only local identities above 25% (E-value $10^{-10}$) are shown. Nodes are colored per protein family.

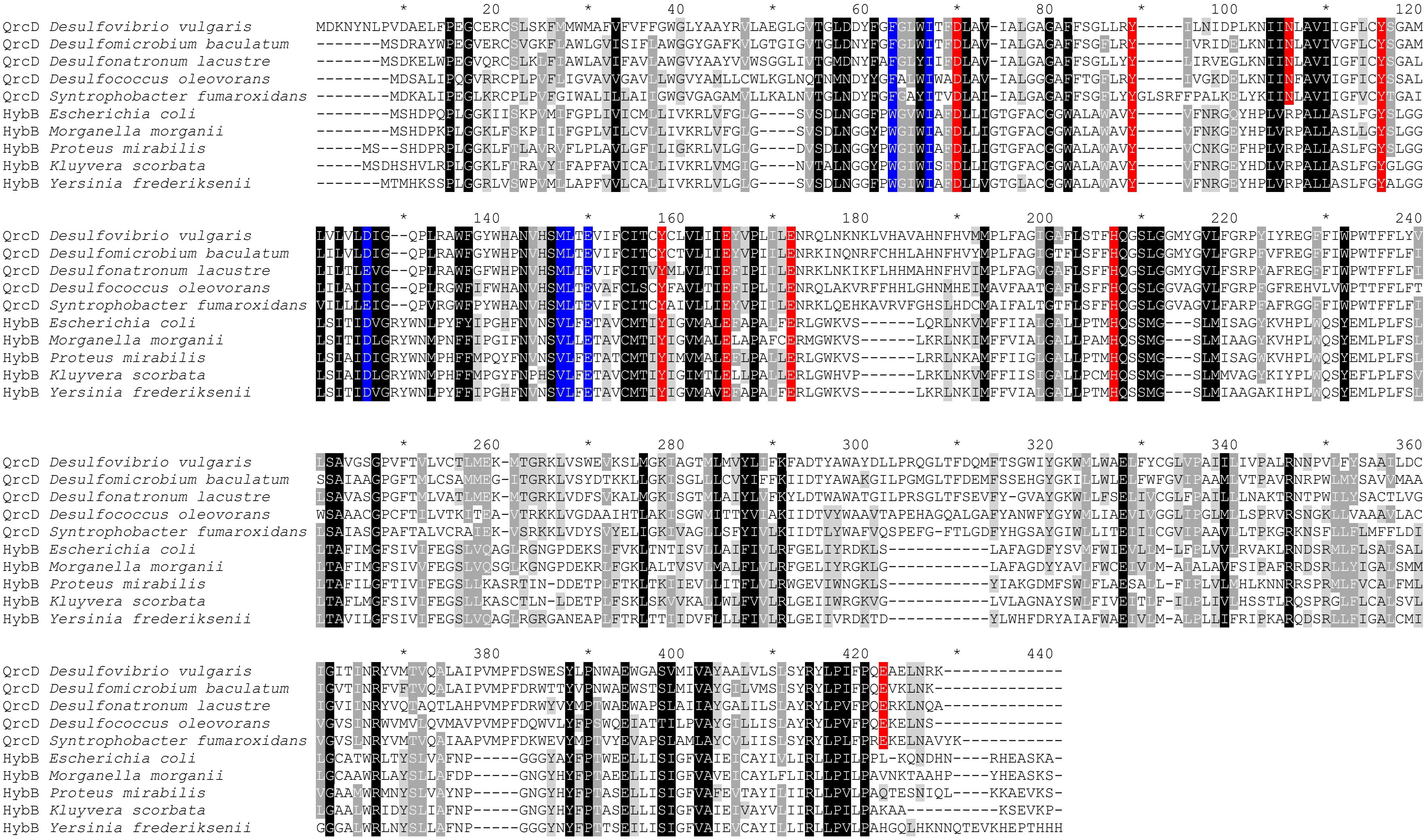


**Suplementary Fig. 13. QrcD and HybB multiple sequence alignment.** Sequences were downloaded from the Joint Genome Institute (https://jgi.doe.gov/) and the aligned with ClustalX 2.1. Conserved residues for the QrcD predicted quinone binding site and proton pathway are coloured in blue and red, respectively.

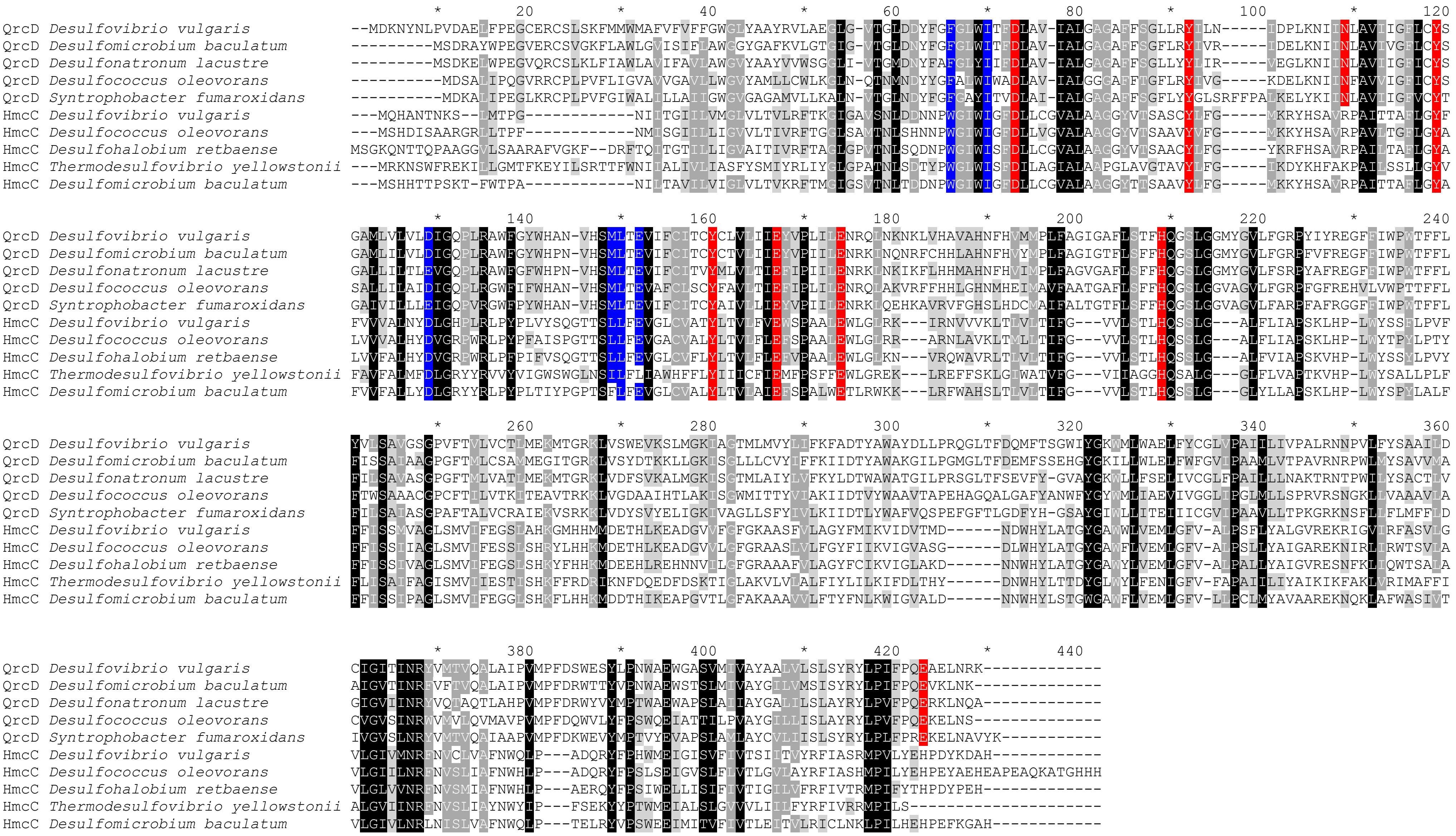


**Supplementary Fig. 14. QrcD and HmcC multiple sequence alignment.** Sequences were downloaded from the Joint Genome Institute (https://jgi.doe.gov/) and the aligned with ClustalX 2.1 program. Conserved residues for the QrcD predicted quinone binding site and proton pathway are coloured in blue and red, respectively.
