# Supplementary material for: An electrogenic redox loop in sulfate reduction reveals a likely widespread mechanism of energy conservation
Source: Nat Commun. 2018 Dec 21;9:5448. doi: 10.1038/s41467-018-07839-x (PMC6303296; doi:10.1038/s41467-018-07839-x)
Supplement: Supplementary file 3 — Description of Additional Supplementary Files [file 41467_2018_7839_MOESM3_ESM.pdf]

Description of Additional Supplementary Files

File Name: Supplementary Data 1
Description: Mass spectrometry data for purified QrcABCD complex

File Name: Supplementary Data 2
Description: Mass spectrometry data for QrcABCD complex extracted from liposomes
