# Supplementary material for: An electrogenic redox loop in sulfate reduction reveals a likely widespread mechanism of energy conservation
Source: Nat Commun. 2018 Dec 21;9:5448. doi: 10.1038/s41467-018-07839-x (PMC6303296; doi:10.1038/s41467-018-07839-x)
Supplement: Supplementary file 4 — Supplementary Data 1 [file 41467_2018_7839_MOESM4_ESM.pdf]

QrcABCD as-isolated

Sample Type: Identification
Cys. Alkylation: Iodoacetamide
Digestion: Trypsin
Instrument: TripleTOF 6600
Special Factors: Gel-based ID
Species:
ID Focus: Biological modifications
Database: uniprot-desulfovibrio-filtered-reviewed.fasta
Search Effort: Thorough
FDR Analysis: Yes
User Modified Parameter Files: Yes

Total	% Cov (9!	Accession #	Name	Species	Peptides(95%)
176.37	93.6	sp|Q72E84|QRCB_DESVH	Menaquinone reductase, molybdopterin-binding-like subunit OS=Desulfovibrio vulgaris (strai	DESVH	1293
63.72	82	sp|Q72E85|QRCC_DESVH	Menaquinone reductase, iron-sulfur cluster-binding subunit OS=Desulfovibrio vulgaris (strain	DESVH	443
23.14	22.9	sp|Q72E86|QRCD_DESVH	Menaquinone reductase, integral membrane subunit OS=Desulfovibrio vulgaris (strain Hilden	DESVH	116
41.24	59.5	sp|Q727D5|EFTU_DESVH	Elongation factor Tu OS=Desulfovibrio vulgaris (strain Hildenborough / ATCC 29579 / DSM 64	DESVH	111
44.73	68.1	sp|Q72E04|ATPB_DESVH	ATP synthase subunit beta OS=Desulfovibrio vulgaris (strain Hildenborough / ATCC 29579 / D!	DESVH	80
45.64	54.8	sp|Q72E02|ATPA_DESVH	ATP synthase subunit alpha OS=Desulfovibrio vulgaris (strain Hildenborough / ATCC 29579 / I	DESVH	77
75.53	55	sp|Q72DV4|SECA_DESVH	Protein translocase subunit SecA OS=Desulfovibrio vulgaris (strain Hildenborough / ATCC 295	DESVH	71
61.66	64.7	sp|Q72CI3|EFG_DESVH	Elongation factor G OS=Desulfovibrio vulgaris (strain Hildenborough / ATCC 29579 / DSM 644	DESVH	58
48.25	63.1	sp|Q72AL6|CH60_DESVH	60 kDa chaperonin OS=Desulfovibrio vulgaris (strain Hildenborough / ATCC 29579 / DSM 644	DESVH	54
53.53	57.4	sp|Q726S7|PTA_DESVH	Phosphate acetyltransferase OS=Desulfovibrio vulgaris (strain Hildenborough / ATCC 29579 /	DESVH	50
31.36	44.2	sp|P45574|DSVA_DESVH	Sulfite reductase, dissimilatory-type subunit alpha OS=Desulfovibrio vulgaris (strain Hildenbor	DESVH	43
34.33	67.9	sp|Q72DQ6|EFTS_DESVH	Elongation factor Ts OS=Desulfovibrio vulgaris (strain Hildenborough / ATCC 29579 / DSM 64	DESVH	40
40.06	41.2	sp|Q728R5|SYT_DESVH	Threonine--tRNA ligase OS=Desulfovibrio vulgaris (strain Hildenborough / ATCC 29579 / DSM	DESVH	37
36.25	55.8	sp|Q72CT0|GLYA_DESVH	Serine hydroxymethyltransferase OS=Desulfovibrio vulgaris (strain Hildenborough / ATCC 295	DESVH	37
33.27	38.2	sp|Q72BL2|PYRG_DESVH	CTP synthase OS=Desulfovibrio vulgaris (strain Hildenborough / ATCC 29579 / DSM 644 / NCII	DESVH	34
15.42	62.9	sp|P0DOV3|QRCA_DESVH	Menaquinone reductase, multiheme cytochrome c subunit OS=Desulfovibrio vulgaris (strain H	DESVH	34
55.88	28.3	sp|Q727C7|RPOB_DESVH	DNA-directed RNA polymerase subunit beta OS=Desulfovibrio vulgaris (strain Hildenborough ,	DESVH	33
23.15	46.2	sp|Q72CF5|RS4_DESVH	30S ribosomal protein S4 OS=Desulfovibrio vulgaris (strain Hildenborough / ATCC 29579 / DSI	DESVH	33

48.22	43.9	sp|Q72AW6|CLPB_DESVH	Chaperone protein ClpB OS=Desulfovibrio vulgaris (strain Hildenborough / ATCC 29579 / DSM	DESVH	32
40.03	30.9	sp|Q72AR5|SYI_DESVH	Isoleucine--tRNA ligase OS=Desulfovibrio vulgaris (strain Hildenborough / ATCC 29579 / DSM	DESVH	32
38.42	46.2	sp|Q72DW8|DNAK_DESVH	Chaperone protein DnaK OS=Desulfovibrio vulgaris (strain Hildenborough / ATCC 29579 / DSM	DESVH	32
46.25	48.8	sp|Q728G0|HTPG_DESVH	Chaperone protein HtpG OS=Desulfovibrio vulgaris (strain Hildenborough / ATCC 29579 / DSM	DESVH	31
18	55.6	sp|Q72CF3|RL17_DESVH	50S ribosomal protein L17 OS=Desulfovibrio vulgaris (strain Hildenborough / ATCC 29579 / DS	DESVH	31
47.22	33.7	sp|Q728S0|SYFB_DESVH	Phenylalanine--tRNA ligase beta subunit OS=Desulfovibrio vulgaris (strain Hildenborough / AT	DESVH	27
43.02	37.4	sp|Q72E47|SYV_DESVH	Valine--tRNA ligase OS=Desulfovibrio vulgaris (strain Hildenborough / ATCC 29579 / DSM 644	DESVH	27
23.92	40.5	sp|Q72D53|YIDC_DESVH	Membrane protein insertase YidC OS=Desulfovibrio vulgaris (strain Hildenborough / ATCC 295	DESVH	25
15.44	47.9	sp|Q728T4|RL13_DESVH	50S ribosomal protein L13 OS=Desulfovibrio vulgaris (strain Hildenborough / ATCC 29579 / DS	DESVH	25
12.54	19.4	sp|T2G6Z9|APRA_DESGG	Adenylylsulfate reductase subunit alpha OS=Desulfovibrio gigas (strain ATCC 19364 / DSM 13	DESGG	25
33.86	44.5	sp|Q72D86|GUAA_DESVH	GMP synthase [glutamine-hydrolyzing] OS=Desulfovibrio vulgaris (strain Hildenborough / ATC	DESVH	23
24.21	40	sp|Q72C59|GCSPA_DESVH	Probable glycine dehydrogenase (decarboxylating) subunit 1 OS=Desulfovibrio vulgaris (strain	DESVH	23
23.41	56	sp|Q72E03|ATPG_DESVH	ATP synthase gamma chain OS=Desulfovibrio vulgaris (strain Hildenborough / ATCC 29579 / D	DESVH	23
11.92	55.6	sp|Q72CG6|RS8_DESVH	30S ribosomal protein S8 OS=Desulfovibrio vulgaris (strain Hildenborough / ATCC 29579 / DSI	DESVH	23
3.39	10	sp|P94692|POR_DESAF	Pyruvate synthase OS=Desulfovibrio africanus OX=873 GN=por PE=1 SV=1	DESAF	23
34.02	19.4	sp|Q727C6|RPOC_DESVH	DNA-directed RNA polymerase subunit beta' OS=Desulfovibrio vulgaris (strain Hildenborough	DESVH	22
22.43	52.2	sp|P45575|DSVB_DESVH	Sulfite reductase, dissimilatory-type subunit beta OS=Desulfovibrio vulgaris (strain Hildenborc	DESVH	22
22.96	50	sp|Q72DQ5|RS2_DESVH	30S ribosomal protein S2 OS=Desulfovibrio vulgaris (strain Hildenborough / ATCC 29579 / DSI	DESVH	21
23.06	38	sp|Q72C23|HEM1_DESVH	Glutamyl-tRNA reductase OS=Desulfovibrio vulgaris (strain Hildenborough / ATCC 29579 / DS	DESVH	21
13.88	68.6	sp|P61940|RISB_DESVH	6,7-dimethyl-8-ribityllumazine synthase OS=Desulfovibrio vulgaris (strain Hildenborough / AT	DESVH	19
31	38.7	sp|Q72CD3|DXS_DESVH	1-deoxy-D-xylulose-5-phosphate synthase OS=Desulfovibrio vulgaris (strain Hildenborough / A	DESVH	18
27.61	26.9	sp|P61700|SYA_DESVH	Alanine--tRNA ligase OS=Desulfovibrio vulgaris (strain Hildenborough / ATCC 29579 / DSM 64	DESVH	17
17.83	38.7	sp|Q72D35|OTC_DESVH	Ornithine carbamoyltransferase OS=Desulfovibrio vulgaris (strain Hildenborough / ATCC 2957	DESVH	17
17.6	13.2	sp|B8DRH3|SECA_DESVM	Protein translocase subunit SecA OS=Desulfovibrio vulgaris (strain Miyazaki F / DSM 19637) O	DESVM	17
28.12	18.8	sp|Q72ER1|IF2_DESVH	Translation initiation factor IF-2 OS=Desulfovibrio vulgaris (strain Hildenborough / ATCC 2957	DESVH	16
26.17	44.4	sp|Q725K9|PURA_DESVH	Adenylosuccinate synthetase OS=Desulfovibrio vulgaris (strain Hildenborough / ATCC 29579 /	DESVH	16
22.79	27.5	sp|Q725Q7|SYDND_DESVH	Aspartate--tRNA(Asp/Asn) ligase OS=Desulfovibrio vulgaris (strain Hildenborough / ATCC 295	DESVH	16
21.01	42.3	sp|Q72CS5|PLSX_DESVH	Phosphate acyltransferase OS=Desulfovibrio vulgaris (strain Hildenborough / ATCC 29579 / DS	DESVH	16
18.31	66.3	sp|Q72CG3|RS5_DESVH	30S ribosomal protein S5 OS=Desulfovibrio vulgaris (strain Hildenborough / ATCC 29579 / DSI	DESVH	16
23.22	46.6	sp|Q72B14|MTAD_DESVH	5-methylthioadenosine/S-adenosylhomocysteine deaminase OS=Desulfovibrio vulgaris (strair	DESVH	15
12.01	42.4	sp|Q72CS6|FABH_DESVH	3-oxoacyl-[acyl-carrier-protein] synthase 3 OS=Desulfovibrio vulgaris (strain Hildenborough /	DESVH	15
25.53	49	sp|Q728D5|GLMU_DESVH	Bifunctional protein GlmU OS=Desulfovibrio vulgaris (strain Hildenborough / ATCC 29579 / DS	DESVH	14
23.96	38	sp|Q726H4|GLPK_DESVH	Glycerol kinase OS=Desulfovibrio vulgaris (strain Hildenborough / ATCC 29579 / DSM 644 / N	DESVH	14

<table>
<tr><td>19.98</td><td>40.8</td><td>sp|Q72FW5|POTA_DESVH</td><td>Spermidine/putrescine import ATP-binding protein PotA OS=Desulfovibrio vulgaris (strain Hilc</td><td>DESVH</td><td>14</td></tr>
<tr><td>17.55</td><td>42.9</td><td>sp|Q726J4|CARA_DESVH</td><td>Carbamoyl-phosphate synthase small chain OS=Desulfovibrio vulgaris (strain Hildenborough /</td><td>DESVH</td><td>14</td></tr>
<tr><td>23.1</td><td>33.8</td><td>sp|Q72BQ5|SYC_DESVH</td><td>Cysteine--tRNA ligase OS=Desulfovibrio vulgaris (strain Hildenborough / ATCC 29579 / DSM 6</td><td>DESVH</td><td>13</td></tr>
<tr><td>19.53</td><td>54.3</td><td>sp|Q72CH4|RS3_DESVH</td><td>30S ribosomal protein S3 OS=Desulfovibrio vulgaris (strain Hildenborough / ATCC 29579 / DSI</td><td>DESVH</td><td>13</td></tr>
<tr><td>18.47</td><td>16.4</td><td>sp|Q72CE6|LON_DESVH</td><td>Lon protease OS=Desulfovibrio vulgaris (strain Hildenborough / ATCC 29579 / DSM 644 / NCII</td><td>DESVH</td><td>12</td></tr>
<tr><td>17.26</td><td>33.8</td><td>sp|P33389|HMC2_DESVH</td><td>Protein DVU_0535 OS=Desulfovibrio vulgaris (strain Hildenborough / ATCC 29579 / DSM 644</td><td>DESVH</td><td>12</td></tr>
<tr><td>12.97</td><td>29</td><td>sp|P62412|PGK_DESVH</td><td>Phosphoglycerate kinase OS=Desulfovibrio vulgaris (strain Hildenborough / ATCC 29579 / DSI</td><td>DESVH</td><td>12</td></tr>
<tr><td>22.67</td><td>29.9</td><td>sp|Q729V4|GLGA_DESVH</td><td>Glycogen synthase OS=Desulfovibrio vulgaris (strain Hildenborough / ATCC 29579 / DSM 644</td><td>DESVH</td><td>11</td></tr>
<tr><td>18.36</td><td>32.3</td><td>sp|Q726S6|ACKA_DESVH</td><td>Acetate kinase OS=Desulfovibrio vulgaris (strain Hildenborough / ATCC 29579 / DSM 644 / NC</td><td>DESVH</td><td>11</td></tr>
<tr><td>18.27</td><td>28.2</td><td>sp|Q725Q1|ILVD_DESVH</td><td>Dihydroxy-acid dehydratase OS=Desulfovibrio vulgaris (strain Hildenborough / ATCC 29579 / I</td><td>DESVH</td><td>11</td></tr>
<tr><td>18.07</td><td>30.5</td><td>sp|Q725I1|GSA_DESVH</td><td>Glutamate-1-semialdehyde 2,1-aminomutase OS=Desulfovibrio vulgaris (strain Hildenborougl</td><td>DESVH</td><td>11</td></tr>
<tr><td>15.34</td><td>42.6</td><td>sp|Q72AQ6|PHNC_DESVH</td><td>Phosphonates import ATP-binding protein PhnC OS=Desulfovibrio vulgaris (strain Hildenborou</td><td>DESVH</td><td>11</td></tr>
<tr><td>6.01</td><td>24.6</td><td>sp|Q30Z56|RS8_DESAG</td><td>30S ribosomal protein S8 OS=Desulfovibrio alaskensis (strain G20) OX=207559 GN=rpsH PE=3</td><td>DESAG</td><td>11</td></tr>
<tr><td>17.36</td><td>37.2</td><td>sp|Q72DH6|SYY_DESVH</td><td>Tyrosine--tRNA ligase OS=Desulfovibrio vulgaris (strain Hildenborough / ATCC 29579 / DSM 6</td><td>DESVH</td><td>10</td></tr>
<tr><td>15.13</td><td>35.1</td><td>sp|Q728U8|MURG_DESVH</td><td>UDP-N-acetylglucosamine--N-acetylmuramyl-(pentapeptide) pyrophosphoryl-undecaprenol N</td><td>DESVH</td><td>10</td></tr>
<tr><td>14.31</td><td>66.5</td><td>sp|Q72DH1|RL9_DESVH</td><td>50S ribosomal protein L9 OS=Desulfovibrio vulgaris (strain Hildenborough / ATCC 29579 / DSI</td><td>DESVH</td><td>10</td></tr>
<tr><td>13.6</td><td>22.7</td><td>sp|P61522|ASSY_DESVH</td><td>Argininosuccinate synthase OS=Desulfovibrio vulgaris (strain Hildenborough / ATCC 29579 / D</td><td>DESVH</td><td>10</td></tr>
<tr><td>9.78</td><td>26.3</td><td>sp|Q726X7|PSD_DESVH</td><td>Phosphatidylserine decarboxylase proenzyme OS=Desulfovibrio vulgaris (strain Hildenborougl</td><td>DESVH</td><td>10</td></tr>
</table>

ID statistics table

Unused (Conf) Cutoff	Proteins Detected	Proteins Before Grouping	Distinct Peptides	Spectra Identified	% Total Spectra
>2.0 (99)	190	459	6106	19158	20.8
>1.3 (95)	214	536	6177	19293	21
>0.47 (66)	236	595	6249	19418	21.1
Cutoff Applied: >0.10 (20%	269	663	6355	19597	21.3
