# Supplementary material for: An electrogenic redox loop in sulfate reduction reveals a likely widespread mechanism of energy conservation
Source: Nat Commun. 2018 Dec 21;9:5448. doi: 10.1038/s41467-018-07839-x (PMC6303296; doi:10.1038/s41467-018-07839-x)
Supplement: Supplementary file 5 — Supplementary Data 2 [file 41467_2018_7839_MOESM5_ESM.pdf]

QrcABCD sample after extraction from liposomes

**Sample Type:**	Identification
**Cys. Alkylation:**	Iodoacetamide
**Digestion:**	Trypsin
**Instrument:**	TripleTOF 6600
**Special Factors:**	Gel-based ID
**Species:**	
**ID Focus:**	Biological modifications
**Database:**	uniprot-desulfovibrio-filtered-reviewed.fasta
**Search Effort:**	Thorough
**FDR Analysis:**	Yes
**User Modified Parameter Files**	Yes

Total	% Cov (9!	Accession #	Name	Species	Peptides(95%)
168.75	93.6	sp|Q72E84|QRCB_DESVH	Menaquinone reductase, molybdopterin-binding-like subunit OS=Desulfovibrio vulgaris (strai	DESVH	1616
52.94	74.1	sp|Q72E85|QRCC_DESVH	Menaquinone reductase, iron-sulfur cluster-binding subunit OS=Desulfovibrio vulgaris (strain	DESVH	352
17.55	19.6	sp|Q72E86|QRCD_DESVH	Menaquinone reductase, integral membrane subunit OS=Desulfovibrio vulgaris (strain Hilden	DESVH	109
47.09	51.8	sp|Q72E02|ATPA_DESVH	ATP synthase subunit alpha OS=Desulfovibrio vulgaris (strain Hildenborough / ATCC 29579 / D	DESVH	89
72.95	55.1	sp|Q72DV4|SECA_DESVH	Protein translocase subunit SecA OS=Desulfovibrio vulgaris (strain Hildenborough / ATCC 295	DESVH	78
38.64	68.1	sp|Q72E04|ATPB_DESVH	ATP synthase subunit beta OS=Desulfovibrio vulgaris (strain Hildenborough / ATCC 29579 / D	DESVH	73
11.59	42.9	sp|P0DOV3|QRCA_DESVH	Menaquinone reductase, multiheme cytochrome c subunit OS=Desulfovibrio vulgaris (strain H	DESVH	28
23.41	40.5	sp|Q72D53|YIDC_DESVH	Membrane protein insertase YidC OS=Desulfovibrio vulgaris (strain Hildenborough / ATCC 29	DESVH	27
23.21	55.7	sp|Q72E03|ATPG_DESVH	ATP synthase gamma chain OS=Desulfovibrio vulgaris (strain Hildenborough / ATCC 29579 / D	DESVH	23
32.4	61	sp|Q727D5|EFTU_DESVH	Elongation factor Tu OS=Desulfovibrio vulgaris (strain Hildenborough / ATCC 29579 / DSM 64	DESVH	94
62.11	64.3	sp|Q72CI3|EFG_DESVH	Elongation factor G OS=Desulfovibrio vulgaris (strain Hildenborough / ATCC 29579 / DSM 644	DESVH	71
54.34	58.8	sp|Q726S7|PTA_DESVH	Phosphate acetyltransferase OS=Desulfovibrio vulgaris (strain Hildenborough / ATCC 29579 /	DESVH	51
40.87	60.5	sp|Q72AL6|CH60_DESVH	60 kDa chaperonin OS=Desulfovibrio vulgaris (strain Hildenborough / ATCC 29579 / DSM 644	DESVH	43
37.82	36.2	sp|Q728R5|SYT_DESVH	Threonine--tRNA ligase OS=Desulfovibrio vulgaris (strain Hildenborough / ATCC 29579 / DSM	DESVH	37
28.68	40.3	sp|P45574|DSVA_DESVH	Sulfite reductase, dissimilatory-type subunit alpha OS=Desulfovibrio vulgaris (strain Hildenbor	DESVH	32
40.86	29.1	sp|Q72AR5|SYI_DESVH	Isoleucine--tRNA ligase OS=Desulfovibrio vulgaris (strain Hildenborough / ATCC 29579 / DSM	DESVH	31

55.26	25.7	sp|Q727C7|RPOB_DESVH	DNA-directed RNA polymerase subunit beta OS=Desulfovibrio vulgaris (strain Hildenborough /	DESVH	30
29.59	65.9	sp|Q72DQ6|EFTS_DESVH	Elongation factor Ts OS=Desulfovibrio vulgaris (strain Hildenborough / ATCC 29579 / DSM 64	DESVH	29
16.73	55.6	sp|Q72CF3|RL17_DESVH	50S ribosomal protein L17 OS=Desulfovibrio vulgaris (strain Hildenborough / ATCC 29579 / DS	DESVH	29
32.43	49.3	sp|Q72CT0|GLYA_DESVH	Serine hydroxymethyltransferase OS=Desulfovibrio vulgaris (strain Hildenborough / ATCC 295	DESVH	28
26.97	31.6	sp|Q72BL2|PYRG_DESVH	CTP synthase OS=Desulfovibrio vulgaris (strain Hildenborough / ATCC 29579 / DSM 644 / NCII	DESVH	28
43.85	42.9	sp|Q728G0|HTPG_DESVH	Chaperone protein HtpG OS=Desulfovibrio vulgaris (strain Hildenborough / ATCC 29579 / DSN	DESVH	26
40.19	36.2	sp|Q728S0|SYFB_DESVH	Phenylalanine--tRNA ligase beta subunit OS=Desulfovibrio vulgaris (strain Hildenborough / AT	DESVH	26
45.55	36.3	sp|Q72AW6|CLPB_DESVH	Chaperone protein ClpB OS=Desulfovibrio vulgaris (strain Hildenborough / ATCC 29579 / DSM	DESVH	25
40.82	31	sp|Q72E47|SYV_DESVH	Valine--tRNA ligase OS=Desulfovibrio vulgaris (strain Hildenborough / ATCC 29579 / DSM 644	DESVH	25
22.21	42.3	sp|Q72CF5|RS4_DESVH	30S ribosomal protein S4 OS=Desulfovibrio vulgaris (strain Hildenborough / ATCC 29579 / DSI	DESVH	25
12.06	23	sp|T2G6Z9|APRA_DESGG	Adenylylsulfate reductase subunit alpha OS=Desulfovibrio gigas (strain ATCC 19364 / DSM 13	DESGG	24
38.54	20.1	sp|Q727C6|RPOC_DESVH	DNA-directed RNA polymerase subunit beta' OS=Desulfovibrio vulgaris (strain Hildenborough	DESVH	23
32.12	46.6	sp|Q72D86|GUAA_DESVH	GMP synthase [glutamine-hydrolyzing] OS=Desulfovibrio vulgaris (strain Hildenborough / ATC	DESVH	22
30.45	37.1	sp|Q72DW8|DNAK_DESVH	Chaperone protein DnaK OS=Desulfovibrio vulgaris (strain Hildenborough / ATCC 29579 / DSN	DESVH	22
22.82	50	sp|Q72DQ5|RS2_DESVH	30S ribosomal protein S2 OS=Desulfovibrio vulgaris (strain Hildenborough / ATCC 29579 / DSI	DESVH	21
2.57	14.2	sp|P94692|POR_DESAF	Pyruvate synthase OS=Desulfovibrio africanus OX=873 GN=por PE=1 SV=1	DESAF	21
26.37	33.7	sp|Q72CD3|DXS_DESVH	1-deoxy-D-xylulose-5-phosphate synthase OS=Desulfovibrio vulgaris (strain Hildenborough /	DESVH	18
21.54	31.2	sp|Q72C59|GCSPA_DESVH	Probable glycine dehydrogenase (decarboxylating) subunit 1 OS=Desulfovibrio vulgaris (strain	DESVH	18
18.69	35.2	sp|P45575|DSVB_DESVH	Sulfite reductase, dissimilatory-type subunit beta OS=Desulfovibrio vulgaris (strain Hildenbor	DESVH	17
17.86	38.7	sp|Q72D35|OTC_DESVH	Ornithine carbamoyltransferase OS=Desulfovibrio vulgaris (strain Hildenborough / ATCC 2957	DESVH	17
15.46	44.3	sp|Q72CH4|RS3_DESVH	30S ribosomal protein S3 OS=Desulfovibrio vulgaris (strain Hildenborough / ATCC 29579 / DSI	DESVH	17
24.59	23.8	sp|P61700|SYA_DESVH	Alanine--tRNA ligase OS=Desulfovibrio vulgaris (strain Hildenborough / ATCC 29579 / DSM 64	DESVH	16
23.68	36.1	sp|Q72C23|HEM1_DESVH	Glutamyl-tRNA reductase OS=Desulfovibrio vulgaris (strain Hildenborough / ATCC 29579 / DS	DESVH	16
12.55	44.4	sp|Q728T4|RL13_DESVH	50S ribosomal protein L13 OS=Desulfovibrio vulgaris (strain Hildenborough / ATCC 29579 / DS	DESVH	16
19.87	51.3	sp|Q72AQ6|PHNC_DESVH	Phosphonates import ATP-binding protein PhnC OS=Desulfovibrio vulgaris (strain Hildenborou	DESVH	15
17.93	38.7	sp|Q72B14|MTAD_DESVH	5-methylthioadenosine/S-adenosylhomocysteine deaminase OS=Desulfovibrio vulgaris (strair	DESVH	15
12.77	55.6	sp|Q72CG6|RS8_DESVH	30S ribosomal protein S8 OS=Desulfovibrio vulgaris (strain Hildenborough / ATCC 29579 / DSI	DESVH	15
24.49	17.1	sp|Q72AU3|SYGB_DESVH	Glycine--tRNA ligase beta subunit OS=Desulfovibrio vulgaris (strain Hildenborough / ATCC 295	DESVH	14
24.42	15.2	sp|Q72ER1|IF2_DESVH	Translation initiation factor IF-2 OS=Desulfovibrio vulgaris (strain Hildenborough / ATCC 2957	DESVH	14
24	39.8	sp|Q726H4|GLPK_DESVH	Glycerol kinase OS=Desulfovibrio vulgaris (strain Hildenborough / ATCC 29579 / DSM 644 / N	DESVH	14
23.4	34.6	sp|Q72BQ5|SYC_DESVH	Cysteine--tRNA ligase OS=Desulfovibrio vulgaris (strain Hildenborough / ATCC 29579 / DSM 6	DESVH	12
20.39	35.7	sp|Q72CS5|PLSX_DESVH	Phosphate acyltransferase OS=Desulfovibrio vulgaris (strain Hildenborough / ATCC 29579 / DS	DESVH	12
20.25	20.3	sp|Q725Q7|SYDND_DESVH	Aspartate--tRNA(Asp/Asn) ligase OS=Desulfovibrio vulgaris (strain Hildenborough / ATCC 295	DESVH	12

16.15	40.3	sp|Q726J4|CARA_DESVH	Carbamoyl-phosphate synthase small chain OS=Desulfovibrio vulgaris (strain Hildenborough /	DESVH	12
15.3	58.9	sp|Q72CG3|RS5_DESVH	30S ribosomal protein S5 OS=Desulfovibrio vulgaris (strain Hildenborough / ATCC 29579 / DSI	DESVH	12
18.34	30.5	sp|Q725K9|PURA_DESVH	Adenylosuccinate synthetase OS=Desulfovibrio vulgaris (strain Hildenborough / ATCC 29579 /	DESVH	11
15.89	24.8	sp|P61522|ASSY_DESVH	Argininosuccinate synthase OS=Desulfovibrio vulgaris (strain Hildenborough / ATCC 29579 / D	DESVH	11
14.84	27	sp|P33389|HMC2_DESVH	Protein DVU_0535 OS=Desulfovibrio vulgaris (strain Hildenborough / ATCC 29579 / DSM 644	DESVH	11
13.23	43.3	sp|Q72CS6|FABH_DESVH	3-oxoacyl-[acyl-carrier-protein] synthase 3 OS=Desulfovibrio vulgaris (strain Hildenborough /	DESVH	11
18.97	34.3	sp|Q728D5|GLMU_DESVH	Bifunctional protein GlmU OS=Desulfovibrio vulgaris (strain Hildenborough / ATCC 29579 / DS	DESVH	10
16.55	28.8	sp|Q725I1|GSA_DESVH	Glutamate-1-semialdehyde 2,1-aminomutase OS=Desulfovibrio vulgaris (strain Hildenborougl	DESVH	10
14.99	21.7	sp|Q725Q1|ILVD_DESVH	Dihydroxy-acid dehydratase OS=Desulfovibrio vulgaris (strain Hildenborough / ATCC 29579 / I	DESVH	10
14.6	61.7	sp|Q72DH1|RL9_DESVH	50S ribosomal protein L9 OS=Desulfovibrio vulgaris (strain Hildenborough / ATCC 29579 / DSI	DESVH	10
14.01	64.1	sp|P61940|RISB_DESVH	6,7-dimethyl-8-ribityllumazine synthase OS=Desulfovibrio vulgaris (strain Hildenborough / AT	DESVH	10
10.7	40.2	sp|Q72CI0|RL3_DESVH	50S ribosomal protein L3 OS=Desulfovibrio vulgaris (strain Hildenborough / ATCC 29579 / DSI	DESVH	10
5.8	47.6	sp|P45573|DSVC_DESVH	Sulfite reductase, dissimilatory-type subunit gamma OS=Desulfovibrio vulgaris (strain Hildenb	DESVH	10
15.38	35.9	sp|Q72FW5|POTA_DESVH	Spermidine/putrescine import ATP-binding protein PotA OS=Desulfovibrio vulgaris (strain Hilc	DESVH	12

ID statistics table

Unused (	Proteins	Proteins Before Grouping	Distinct Peptides	Spectra Ider	% Total Spectra
>2.0 (99)	185	435	5914	18381	19.5
>1.3 (95)	212	521	5989	18503	19.6
>0.47 (6	234	575	6062	18625	19.7
Cutoff A	266	635	6152	18770	19.9
